# Use of radiometric (^234/238^U and ^228/226^Ra) and mass spectrometry (^87/86^Sr) methods for studies of the stability of groundwater reservoirs in Central Poland

**DOI:** 10.1007/s10967-014-3390-9

**Published:** 2014-08-24

**Authors:** P. Grabowski, H. Bem, R. L. Romer

**Affiliations:** 1 Department of Chemistry, Technical University of Lodz, Zeromskiego Street 116, 90-924 Lodz, Poland; 2 Deutsches GeoForschungsZentrum, Telegrafenberg, 14473 Potsdam, Germany; 3 Present Address: Faculty of Building Engineering, Mechanics, and Petrochemistry, Institute of Chemistry, Warsaw University of Technology, Lukasiewicza Street 17, 09-400 Plock, Poland

**Keywords:** ^228^Ra/^226^Ra activity ratio, ^234^U/^238^U activity ratio, ^87^Sr/^86^Sr isotopic ratio, Thermal groundwater, Isotopic study

## Abstract

The uranium (^234^U/^238^U) and radium (^228^Ra/^226^Ra) activity ratios and ^87^Sr/^86^Sr isotopic ratio in thermal groundwater, subsurface water (groundwater) and river water from Poddebice and Uniejow were determined. The uranium and radium activity ratios and strontium isotopic ratio varied from 0.629 to 1.471, from 0.396 to 4.961 and from 0.708438 to 0.710344, respectively. The results for the thermal groundwater samples showed that the radiometric method together with mass spectrometry stable strontium isotope ratio measurements can be used for underground water transport studies. On the basis of the uranium and radium activity and the strontium isotopic ratio differences in subsurface water (groundwater) and in river water, any possible water influx between these adjacent reservoirs can be observed. The obtained results exclude any water transport from surface and subsurface water to thermal ground water reservoirs in this region of Poland.

## Introduction

In some kinds of environmental samples one can observe small differences in the isotopic ratios for a few elements caused by the continuous production of one of their so-called radiogenic isotopes by radioactive decay (in a geological time scale of 10^6^ years) of accompanying radioactive isotopes of other elements. Changes in the isotopic composition can be used for the observation and interpretation of physicochemical processes, for example, in water–rock systems, and as a source of information on the weathering of rocks and other hydrogeological transformations [1].

The first applications of radiogenic isotopes to weathering processes were based on studies of assessing the impact of chemical weathering on the geochronology of rock and mineral samples [2, 3], as well as the study of the isotope effect during the leaching of rock by acids [4]. The main conclusion of these studies was that weathering influences the occurrence of anomalies in the determination of age by Rb–Sr and U–Pb methods in samples from the same period [5].

One of the naturally occurring rubidium isotopes, ^87^Rb, decays with a very long half-life of 4.88·10^10^ years to the stable ^87^Sr, and its decay influences the natural variability of ^87^Sr/^86^Sr isotopic ratio (IR) in minerals and rocks as well as in natural waters containing ^87^Rb [6]. Originally the strontium isotope ratio was used only in geological and archeological sciences [7, 8], but recently it has also been used in hydrology and studies of subsurface water behavior [9, 10]. The study of Voerkelius et al. [11] shows the change in the ^87^Sr/^86^Sr isotopic ratio in natural mineral water extracted in Europe. In this study, ^87^Sr/^86^Sr ratio values were separated into six groups, which represent the typical values associated with the geological periods. However, in some cases an evaluation of the geological unit formation is not possible solely on the basis of strontium isotopic ratio determinations. This study showed that some samples were taken from the transition geological formation.

Like the strontium isotope ratio method, it is possible to use the U and Th decay series radionuclides to study the interaction of different water components. However, in this case only a few systems of two or more isotopes occur with sufficiently long half-lives for the observation of geochemical processes (i.e. ^238,234^U, ^232,230,228^Th, ^228,226,224^Ra) [12, 13].

In particular, the ^226^Ra radionuclide, with a half-life of 1630 years, can supply important scientific information concerning mechanisms and rates of water–rock interaction and transport of this element in aquifers [14]. The information from such a study can lead to a revised understanding of the controlling water quality steps and can be used to establish better strategies for the use and protection of groundwater reservoir [15]. Similar data can be obtained from uranium ^234^U and ^238^U radionuclide activity ratio [16].

On the basis of geological data for the Central Poland area, the thermal groundwater reservoirs there basically should not exhibit any yearly changes neither in the uranium and radium activity ratios nor the strontium isotopic ratio. In contrast to that such changes can be observed in subsurface water and in river water in this area.

Therefore, it seems to be interesting to compare the radiometric and mass spectrometry methods for evaluation of the chosen isotopic ratios in different types of water samples and observe their changes in the examined water reservoirs.

## Experimental

### Study area

**Fig. 1:**
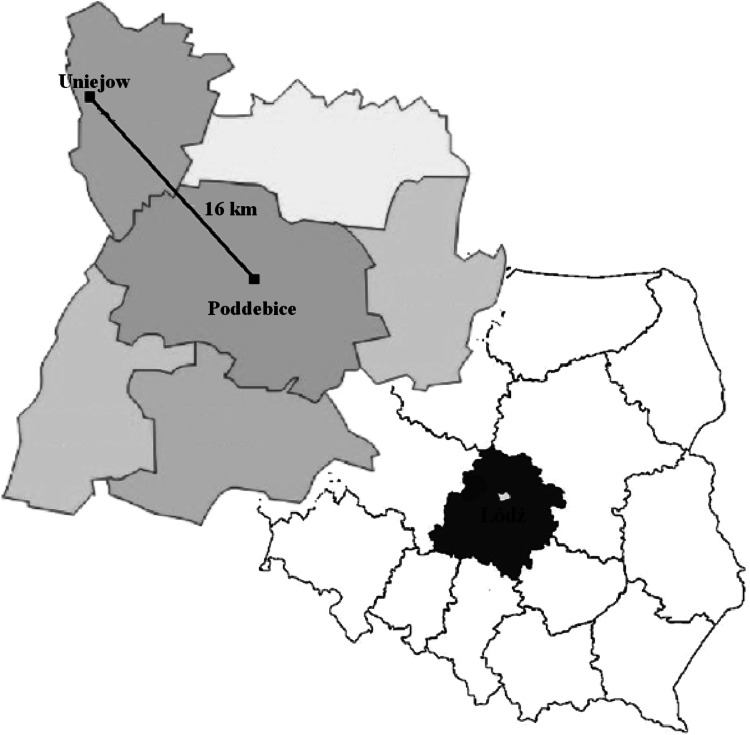
Location of sampling collection sites

Samples of thermal groundwater were collected from their intake station in the vicinity of two cities of Central Poland: Poddebice and Uniejow. The samples of groundwater (70 m below surface) were collected from the drinking water station Poddebice while the river water samples were taken from the Ner river in Poddebice and the Warta river in Uniejow (Fig. 1).

Ten litres of each water sample were collected in polyethylene bottles and, directly after collection, were acidified to pH ≈2. All collected water samples were clear, therefore filtering of the samples was not necessary.

### Radionuclide activity ratio determination

All determined radionuclides (^226,228^Ra, ^234,238^U) were co-precipitated from water samples with hydrated manganese dioxide by a method described elsewhere [17–19].

The activity of ^228^Ra radionuclide via its decay product-^228^Ac was determined using a Canberra spectrometry system with an HPGe detector (relative efficiency −25 %), according to the procedure previously described by us [17, 18]. However, this instrumental method for determination of ^226^Ra via its γ line of 185.6 keV did not ensure a sufficiently low detection limit [20] for environmental water samples (LD ≥ 8.5 mBq/dm^3^) therefore we have used the liquid scintillation method after a 1 month delay followed by extraction of the ^222^Rn from 0.5 dm^3^ dissolved samples (LD = 1.95 mBq/dm^3^) [21].

The activity of uranium isotopes after their deposition on a stainless steel disc were determined by α spectrometry system with a PIPS detector (Canberra Packard). Before measurement, the uranium isotopes were separated on a Dowex 1 × 8 anion exchangeable resin (50–100 mesh, Cl^−^form) by a method described elsewhere [16, 19].

### Strontium isotope ratio determination

The strontium isotope ratio in water samples from Poddebice and Uniejow was determined by thermal ionisation mass spectrometry (TIMS). Sr isotopes were separated using ion-exchange chromatography described elsewhere [22, 23]. Sr was loaded on single tantalum filaments and measured on a TRITON Thermal Ionisation MS using dynamic multi-collection. All Sr isotopic values were normalized to ^86^Sr/^88^Sr = 0.1194.

### Quality assurance of the radiometric method

Due to the fact that the certified reference materials containing measured radionuclides in natural water were not available, such a standard was prepared after mineralisation of about 1 g of soil reference materials—IAEA-327. After mineralisation, the solution was diluted to the same volume which was used for the co-precipitation of radionuclides from the natural water samples (5 dm^3^). All measured radionuclides were co-precipitated with hydrated manganese dioxide and measured in the same way as in natural water samples. The results of their determination are shown in Table 1.

**Table 1:** Chemical recoveries of the determined radionuclides

Nuclide	Reference value (mBq/g)	Measured value (mBq/g)	Recovery (%)
^226^Ra	34.1	34.04 ± 11.51	99.8
^228^Ra(^228^Ac)	38.7	35.21 ± 3.65	91.0
^238^U(^234^Th)	32.8	32.95 ± 5.07	100.5

The obtained results were within the 95 % confidence interval of the recommended or information values for the IAEA-327 standard.

For checking the accuracy of the ^87^Sr/^86^Sr ratio determination, the NBS 987 standard (^87^Sr/^86^Sr = 0.71034 ± 0.00026) was used. The repeated measurement of the NBS 987 Sr standard gave ^87^Sr/^86^Sr = 0.710248 ± 0.000005 (2σ reproducibility for 20 independent analyses). Analytical uncertainties of the Sr measurements are reported as 2σ_m_.

## Results and discussion

### ^228^Ra/^/226^Ra activity ratio in water samples

^228^Ra/^226^Ra activity ratio in groundwater depends on the ratio of its parents’ activity concentrations (^232^Th and ^238^U) in the host rocks, and provide information on the rock–water interaction [24]. Radium isotopes do not show significant fractionation, but nevertheless they are used in geological research for the sake of its long half-life. Radium can penetrate into groundwater as a results of processes such as the decay of dissolved parent radionuclides, alpha recoil, desorption from the surface of water-bearing rock or the dissolution of aquifer rock [15]. In connection with the occurrence of these processes the ^228^Ra/^226^Ra activity ratio can be a useful marker for their characterization in the aquifer rock [25].

**Fig. 2:**
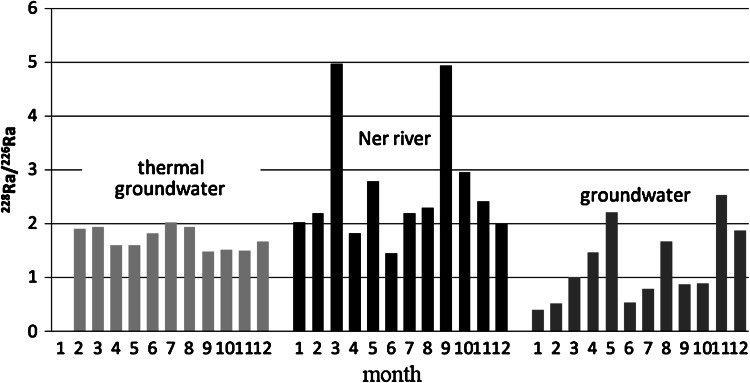
^228^Ra/^226^Ra activity ratio in water samples from Poddebice

**Fig. 3:**
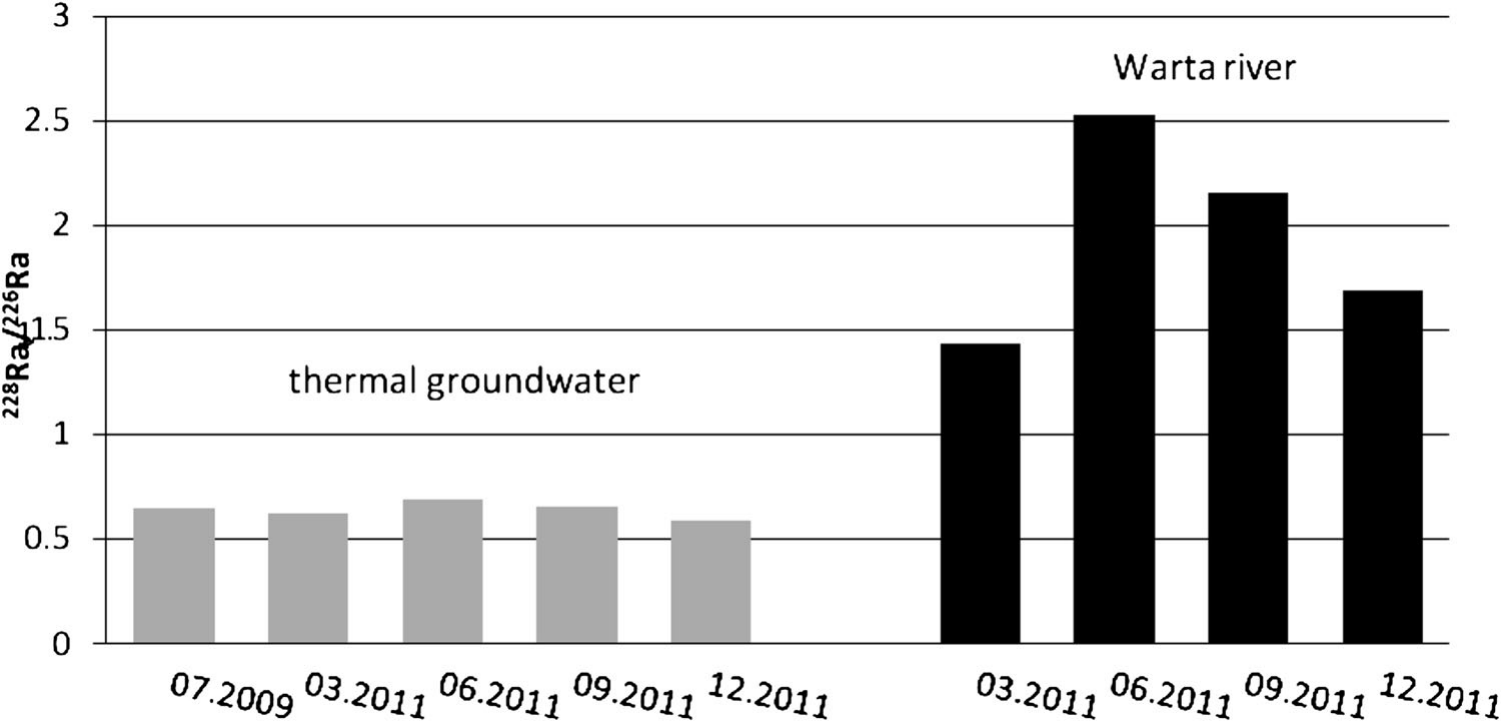
^228^Ra/^226^Ra activity ratio in water samples from Uniejow

In Figs. 2 and 3 the seasonal fluctuations of the ^228/226^Ra activity ratio in different types of water reservoirs from Poddebice and Uniejow are shown, respectively. For the deeply situated (below 1,500 m) thermal groundwater, observed fluctuations are almost negligible with the mean activity ratio of radium isotopes equal to 1.64 and 0.64, respectively. These results show that in Poddebice and Uniejow are two different thermal groundwater type in different rock background in spite of low distance between this two cities (16 km). According to the geological profile in the Poddebice region and information from Geotermia Poddebice Ltd (a company managing the geothermal bore-hole) these are mostly sandstones and arenaceous shale geological formations. Lower ^228^Ra to ^226^Ra activity ratio groundwater reservoir is caused by surface water infiltration from this mostly agricultural area, where fertilizers containing traces of uranium and ^226^Ra are widely used. These results correspond well also to the data obtained by Struchi et al. [15] They reported that for a similar rock background the ^228^Ra/^226^Ra activity ratio ranged from 1.39 to 1.53, whereas for limestone these values were lower and ranged from 0.21 to 0.80.

A thermal groundwater reservoir in the vicinity of the town of Uniejow exists, in contrast to Poddebice’s geothermal water, in limestone formation, therefore the mean activity ratio of radium isotopes, ^228^Ra/^226^Ra, was lower and equal to 0.64 (Fig. 3).

Radium isotopic ratio values in both the Ner and Warta rivers were similar: 2.15 and 1.95, respectively. For this two river the higher values of ^228^Ra/^226^Ra was observed in the March and September. These are caused by solid fallout of the re-suspension of soil which contains slightly higher thorium radionuclide concentrations. These radionuclides can be leached from dust particles which settle on the river basin and can be also transported with rainwater. Similar seasonal fluctuations of the radium isotope ratio (from 0.58 to 2.03) in surface water described by Eikenberg et al. [24].

### U/^238^U isotope ratios

The results concerning uranium activity ratio in thermal groundwater near to Poddebice, and of groundwater and river water from the Ner were described in previous work of the authors [16]. In this work was checked the seasonal fluctuations of this ratio for Uniejow thermal groundwater and the adjacent Warta river.

**Fig. 4:**
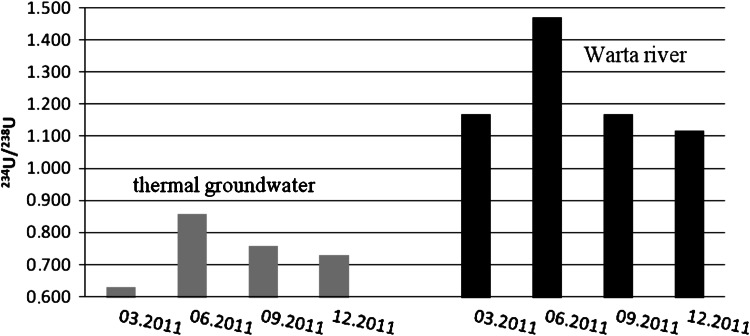
^234^U/^238^U activity ratio in water samples from Uniejow

As in Poddebice, a comparison of the uranium activity ratio in thermal groundwater and in river water from the Warta showed essential differences in these values. The average ratio of ^234^U to ^238^U in the deeply situated thermal groundwater is equal to 0.74 whereas in river water this value slightly exceeds the value of 1 (1.22) (Fig. 4). These results also exclude the possible occurrence of infiltration from surface water to thermal groundwater reservoir in Uniejow region.

### Determination of ^87^Sr do^/86^Sr ratio in the examined water reservoirs

The results of the ^87^Sr/^86^Sr isotopic ratio of measurements in the water samples from Poddebice to Uniejow are shown in Tables 2 and 3. The lowest average strontium isotopic ratio was found in both thermal ground waters and was equal to 0.708492 and 0.708441, respectively. The ^87^Sr/^86^Sr IR in these two waters was close to each other and did not fluctuate during the sampling time.

**Table 2:** ^87^Sr/^86^Sr isotopic ratio in water samples from Poddebice

Date of sample collection	Thermal groundwater	Groundwater	NER river
January 2011	0.708497 ± 4	0.709489 ± 4	0.710344 ± 3
February 2011	0.708492 ± 3	0.709120 ± 2	0.709543 ± 3
March 2011	0.708490 ± 3	0.709480 ± 3	0.709872 ± 4
April 2011	0.708519 ± 3	0.709502 ± 3	0.709187 ± 4
May 2011	0.708496 ± 3	0.709573 ± 3	0.709074 ± 2
June 2011	0.708492 ± 3	0.709510 ± 3	0.708853 ± 2
July 2011	0.708490 ± 3	0.709272 ± 7	0.709075 ± 2
August 2011	0.708486 ± 3	0.709575 ± 5	0.708921 ± 3
September 2011	0.708490 ± 3	0.709480 ± 3	0.708877 ± 4
October 2011	0.708456 ± 3	0.709452 ± 4	0.708994 ± 2
November 2011	0.708494 ± 2	0.709480 ± 4	0.708918 ± 3
December 2011	0.708496 ± 2	0.709515 ± 5	0.708961 ± 3
Average	0.708492	0.709454	0.709218
Standard deviation	0.000014	0.000130	0.000466

**Table 3:** ^87^Sr/^86^Sr isotopic ratio in water samples from Uniejow

Date of sample collection	Thermal groundwater	WARTA river
March 2011	0.708441 ± 4	0.709266 ± 3
June 2011	0.708438 ± 3	0.709325 ± 4
September 2011	0.708441 ± 3	0.709259 ± 3
December 2011	0.708442 ± 3	0.709181 ± 3
Average	0.708441	0.709258
Standard deviation	0.000002	0.000059

A comparison of the ^87^Sr/^86^Sr isotopic ratio in thermal groundwater and subsurface water (tap water) clearly showed a difference between the two water samples from Poddebice. The average strontium isotopic ratios for thermal groundwater and tap water from Poddebice were 0.708492–0.709454, respectively. The difference, already in third place after the decimal point, far exceeds standard deviation values for analytical procedure. It confirms the thesis that subsurface water will not be able to infiltrate the thermal groundwater layers. A different situation was observed for subsurface water and river water from the Ner where ^87^Sr/^86^Sr isotopic ratio were very close and equal to 0.709454 and 0.709218, respectively. Such small changes suggests the possibility infiltration of surface water (river water) to groundwater (subsurface water) in the Poddebice area.

**Fig. 5:**
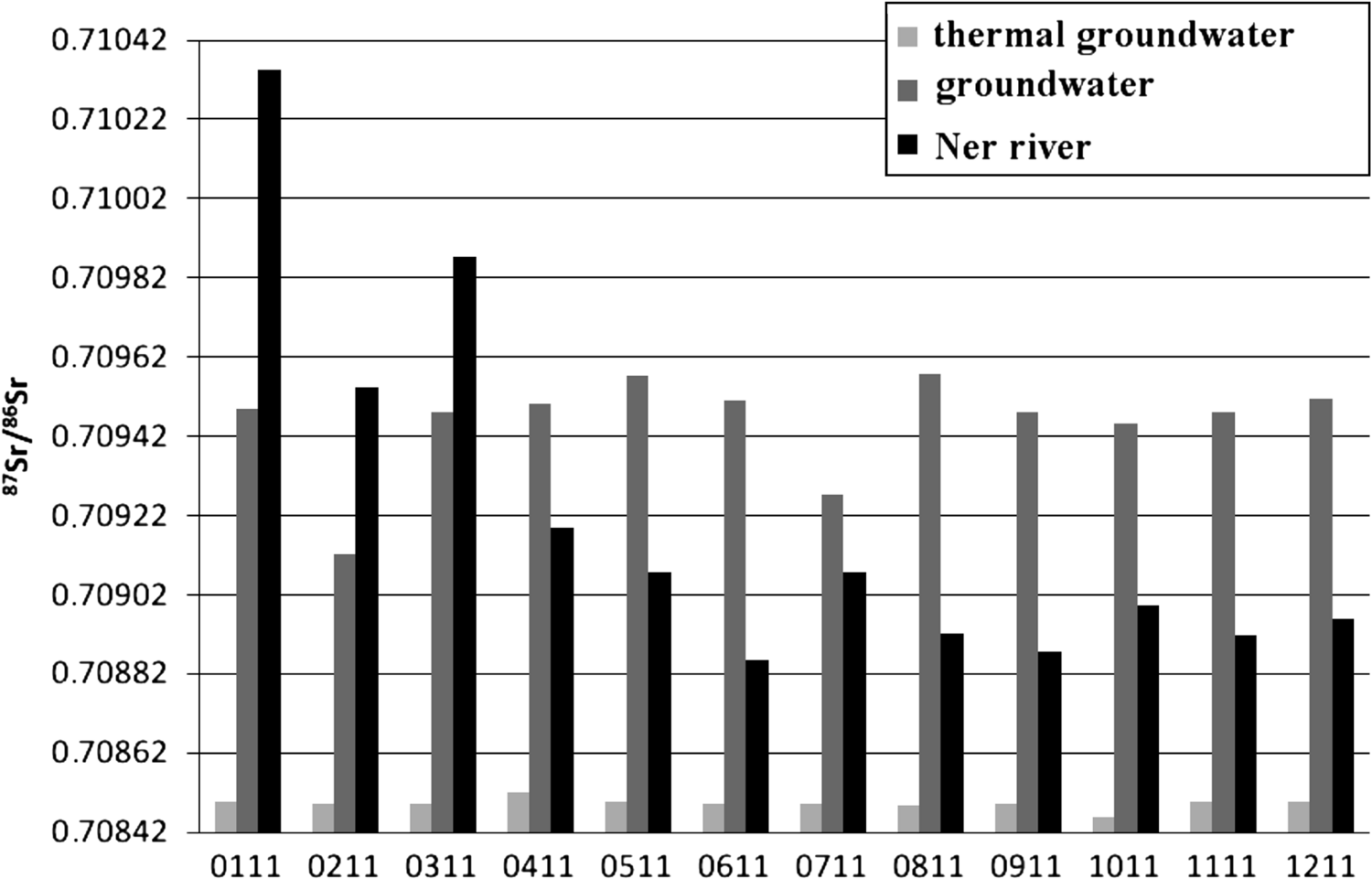
Change of ^87^Sr/^86^Sr isotopic ratio in water samples from Poddebice

**Fig. 6:**
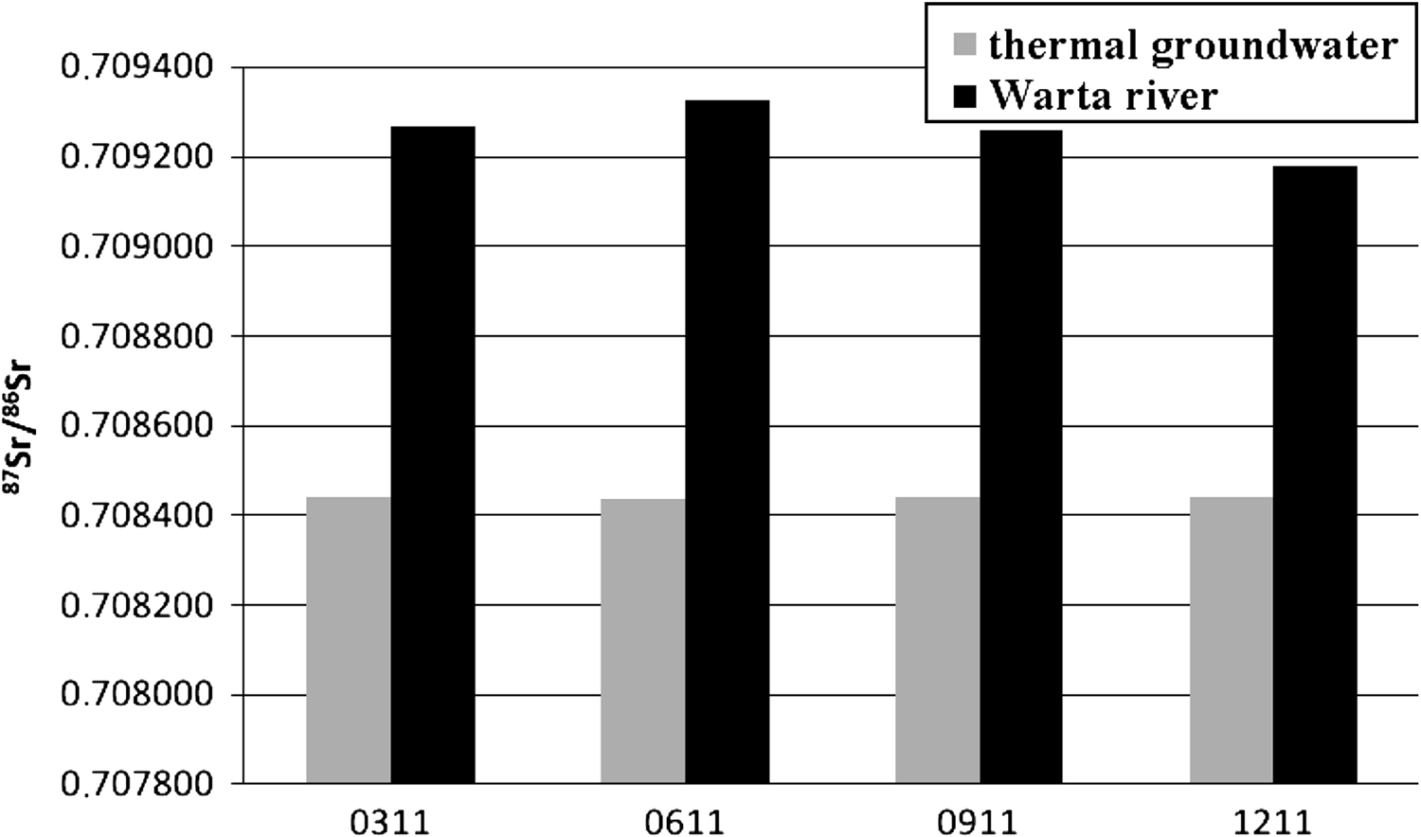
Change of ^87^Sr/^86^Sr isotopic ratio in water samples from Uniejow

These conclusions were confirmed by observation of the monthly fluctuations of strontium isotope ratios in these water samples (Figs. 5, 6) The strontium IR for the Ner river and tap water in Poddebice (supplied from subsurface underground water station) have almost identical shapes. The decrease of the strontium IR is caused by agricultural use of fertilizers. In commonly used agricultural fertilizers the strontium IR changes from 0.703400 to 0.715216 [26]. Higher strontium isotopic ratios are associated with use of such fertilizers as magnesium sulfate, potassium sulfate and multiple fertilizers NPK:15-10-15, which are used in Poland in autumn. Lower strontium isotopic ratios are associated with the use of such fertilizers as magnesium nitrate and ammonium nitrate, which mainly are used in the spring. As was shown on Fig. 5 changes in Sr isotopic ratio induced by using of fertilizer in autumn or spring was not observed directly by its using. This is caused by leaching the soil components by rain and its transport to the river.

Therefore, rainwater drainage from soil to the river basin, particularly for the Ner river, with its relatively short length and flowing mostly through rural areas, plays an important role for changes in the strontium IR. A slightly different situation was observed for the long Warta river, flowing through several big cities (Fig. 6) and such changes are not so clearly seen.

Similar differences in the strontium IR were also observed for surface (river) water by Eikenberg et al. [24]. In the Rhine river the ^87^Sr/^86^Sr isotopic ratio varied from 0.70843 to 0.71798. Such large changes of ^87^Sr/^86^Sr isotopic ratio was also explained by use of agricultural fertilizers and changes in the bedrock chemical composition of the riverbed.

## Conclusions

The application of radiometric and mass spectrometry methods for determination of both radionuclide activity and stable isotope ratios give valuable information concerning mutual transportation between surface, subsurface and deeply situated geothermal water layers.

As expected on the basis of geological data, thermal ground water in Central Poland should not exhibit any seasonal fluctuations either in uranium and radium activity ratios nor the strontium isotopic ratio. It confirms the stability of these reservoirs not influenced by infiltration of the subsurface water. However, on the basis of uranium and radium activity and the strontium isotopic ratios, such infiltration for subsurface water (groundwater) and in river water was confirmed. The obtained results showed that for these purposes the radiometric method can be an alternative solution to the time consuming, expensive but more precise mass spectrometry determinations.

Thermal groundwater from Uniejow is characterized by higher mineralization than thermal groundwater from Poddebice. However, apart from different bedrocks, the close values of the ^87^Sr/^86^Sr isotopic ratio indicate that these water reservoirs were formed in a similar geological period.

Uranium (^234^U/^238^U) and radium (^228^Ra/^226^Ra) activity ratios clearly showed differences in the chemical compositions of the thermal ground water of the Poddebice and Uniejow aquifers due to the different salinity of the water and bedrock compositions. The aquifer in Poddebice is surrounded by sandstone formations, whereas the aquifer in Uniejow is surrounded by clay and limestone layers. Such environments affect the different mineralization of these two water.
